# Application of Artificial Intelligence or machine learning in risk sharing agreements for pharmacotherapy risk management

**DOI:** 10.1515/jib-2023-0014

**Published:** 2023-12-12

**Authors:** Grigory A. Oborotov, Konstantin A. Koshechkin, Yuriy L. Orlov

**Affiliations:** Chair of Information and Internet Technologies, Digital Health Institute, I.M. Sechenov First Moscow State Medical University of the Ministry of Health of the Russian Federation (Sechenov University), Moscow, Russia; Institute of Cytology and Genetics SB RAS, Novosibirsk, Russia; Agrarian and Technological Institute, Peoples’ Friendship University of Russia, Moscow, Russia

**Keywords:** database integration, pharmaceutics, machine learning, risk sharing, managed entry schemes, performance-based risk-sharing arrangements

## Abstract

Applications of Artificial Intelligence in medical informatics solutions risk sharing have social value. At a time of ever-increasing cost for the provision of medicines to citizens, there is a need to restrain the growth of health care costs. The search for computer technologies to stop or slow down the growth of costs acquires a new very important and significant meaning. We discussed the two information technologies in pharmacotherapy and the possibility of combining and sharing them, namely the combination of risk-sharing agreements and Machine Learning, which was made possible by the development of Artificial Intelligence (AI). Neural networks could be used to predict the outcome to reduce the risk factors for treatment. AI-based data processing automation technologies could be also used for risk-sharing agreements automation.

## 1 Introduction

Joint Risk Sharing Agreements (RSA) present breakthrough technological innovations. It can be decisive in terms of the introduction and evaluation of new health technologies [1]. The pharmaceutical and biopharmaceutical industries have advanced in this area over the past decades, having concluded many innovative agreements with customers, government agencies, where the effectiveness of the technology has been proven in clinical trials, but often unresolved issues have arisen about the economic value of the technology or about the clinical comparative effectiveness of the technology in the real world. Payers did not want to bear the financial burden in full of the new technology and did not want to take risks to provide additional financial reimbursement or access to new treatments.

What is often negotiated is the additional collection of real-world data that can track clinical and financial outcomes, and that in real practice there is a link between payment and outcomes [2]. And this can happen at a wide variety of levels thanks to, in fact, agreed contractual agreements in this way. If the manufacturer reaches certain endpoints, it will lead to payment in the future. And in other cases, payments would depend on individual patient outcomes, for example, if a patient underwent a study of a new drug within 30 days, and subsequently benefited from long-term use of the drug, then the manufacturer will be paid according to the price list. But if the test is unsuccessful, the manufacturer will have to reimburse the cost of treatment.

From a broader social perspective [2] RSAs can be seen as an investment in collecting more data to address uncertainties. Any number of stakeholders can be involved in the development, including drug and device manufacturers, public and private payers and insurers, insurance financiers, hospital and physician service providers, central pricing authorities and regional budgets [3–8].

Risk management and Artificial Intelligence (AI) are ideally combined when there is a need to process and evaluate unstructured data. It is assumed that risk managers of various institutions will focus on analytics and loss reduction based on the results of AI performance, which will not waste time managing the risks inherent in operational processes [9].

AI is opening up new horizons, from drug development to improving productivity and improving the outcomes of the entire value chain. Pharmaceutical companies are increasingly introducing automated processes that include data-driven solutions and using predictive analytics tools. A new stage of data analytics includes AI and Machine Learning. AI, which is used in the pharmaceutical industry, is a narrowly focused type of machine intelligence designed to solve specific problems using automated algorithms. The purpose of this technology is to search for hidden patterns and collect information from huge amounts of data in ways that are practically unattainable to humans [10].

Another aspect of AI is based on neural networks that mimic the work of the human brain, while being able to make decisions many times faster and more accurately. Algorithms that are used in machine learning allow software applications to predict results with high accuracy without the need for an explicit description of the logic.

The main advantage for pharmaceutical companies is the ability of AI to reduce the time of testing drugs and obtain permission to enter the market. On the other hand AI is being widely used for new targets discovery and novel drug molecules *in silico* testing. This saves time and money, which, in turn, is already an advantage for payers (which can be state bodies, holders of regional budgets, insurers, chief physicians) since this will affect the lower cost of the drug and the variability in the choice of treatment [10].

It is a well-known fact that more than 80 % of clinical trials do not recruit the required number of patients [11]. This, in turn, has an impact on slowing research and delaying patients’ access to vital drugs [12]. To solve this problem, AI comes to the rescue, capable of searching for patients to participate in tests, also, thanks to the ability to read and analyze unstructured databases, texts (doctor’s records, admission documents, etc.), use data with genetic information for patients, allowing to determine the appropriate patients for research [12, 13].

The goal and challenge of the work was to develop faster and more efficient method for patients to access innovations. Based on the purpose of the study, the following tasks were formulated. Draw the attention of the scientific community to the need to: identification of the new pricing methods based on the advantages that the novel drugs giving; create registries that collect the data necessary for the implementation of RSAs [2]; consider and try to link two promising technologies: technology with the announcement of the separation of risks and AI; analyse different types of risk-sharing agreements; analyse benefits, risks and limitations.

## 2 Methods

Methodology for studying the real use and effectiveness of risk sharing agreements and AI. Scientific search was carried out with the help of the following electronic libraries PubMed MEDLINE is the National Library of Medicine’s (NLM) premier bibliographic database (https://pubmed.ncbi.nlm.nih.gov/), eLibrary.ru (https://elibrary.ru/) is the largest Russian Scientific electronic library, information and analytical portal in the field of science, technology, medicine and education (elibrary.ru), Google Scholar is a search portal for scientific publications considered to be the world’s largest academic search engine, with a coverage rate of up to 90 % of all English-language articles (scholar.google.com) and in addition to the materials of specialized journals. For the purpose of subsequent study, the results of large-scale pharmaco- and clinical-economic studies were first selected. For example, the study by Gonçalves et al. “Risk-sharing agreements, present and future” [1], study by Garrison et al. “Good research practices for measuring drug costs in cost-effectiveness analyses: a societal perspective: the ISPOR Drug Cost Task Force report” [2] etc. Special attention was paid to specialized Internet resources, such as McKinsey & Company, FERMA, TechTarget and Clinical Trials Arena [14–17].

**Figure 1:**
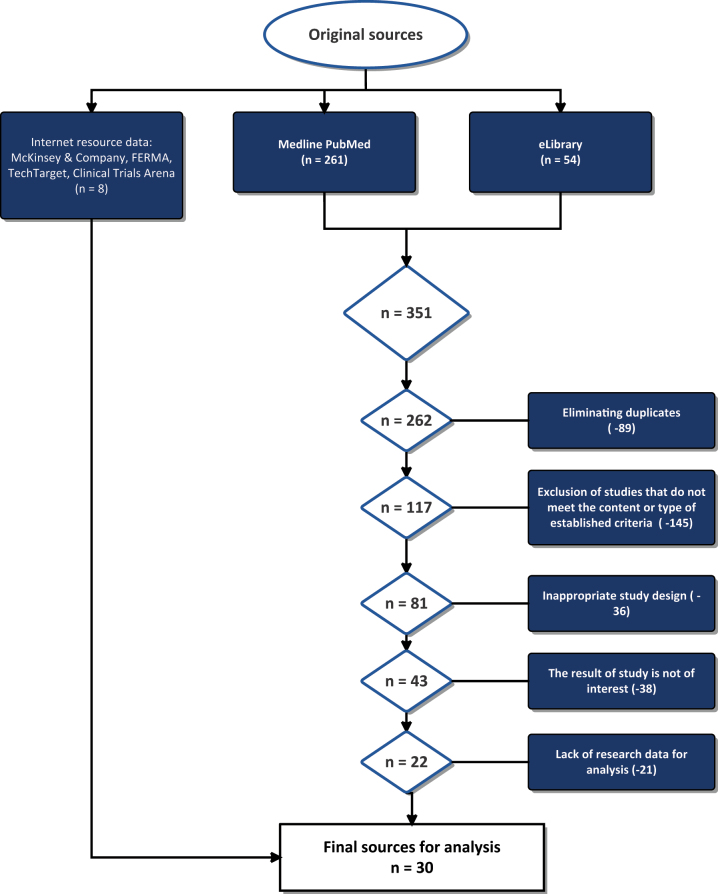
Research inclusion structure.

The first two stages of literature analysis (Figure 1) found 261 records from PubMed and 54 records from eLibrary.ru and others. After checking the headings and annotations, we excluded 89 studies due to duplication and 145 studies due to irrelevance of content or types of publications not meeting predetermined requirements. A total of 81 studies underwent a full-text review, of which 38 studies were excluded because of the result, which was of no interest, and 21 of them were excluded due to insufficient data for analysis. After further exclusion, only 22 studies were selected.

**Figure 2:**
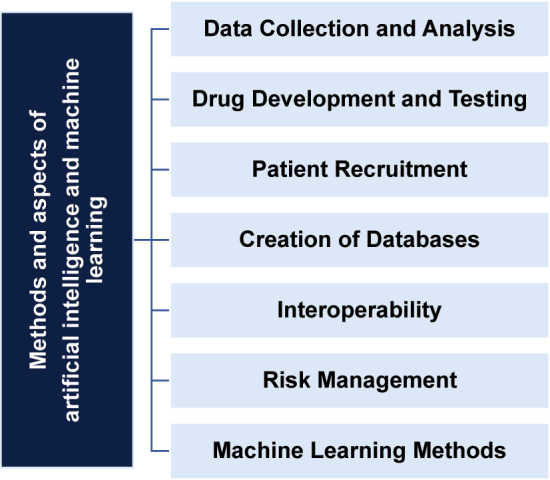
Methods and aspects of artificial intelligence and machine learning.

The research used the following methods and aspects of artificial intelligence and machine learning (Figure 2):

1. Data Collection and Analysis: AI is used to process and evaluate unstructured data, such as clinical records, to track clinical and financial outcomes.
2. Drug Development and Testing: AI accelerates drug testing and regulatory approval processes, reducing time and costs. It is used for new target discovery, in silico testing of drug molecules, and predicting treatment outcomes.
3. Patient Recruitment: AI helps identify and recruit suitable patients for clinical trials by analyzing unstructured databases, including medical records, genetic information, and patient data.
4. Creation of Databases: AI is used to create and maintain databases for risk-sharing agreements, which allows for the efficient collection of data on the use of medicines.
5. Interoperability: Artificial intelligence structures and aggregates data from various sources, but concerns about privacy and data security must be taken into account.
6. Risk Management: Artificial intelligence processes large amounts of data, automates tasks, and provides real-time forecasting capabilities. It improves decision making by providing predictive risk information.
7. Machine Learning Methods: Machine learning features were also used, including artificial neural networks, fuzzy logic, and evolutionary modeling, which are used in medical data analysis and risk management.

## 3 Results

### 3.1 Advantages and disadvantages

There are a number of reasons that justify and encourage the introduction of RSAs, namely increasing the resilience of the health care system without limiting access to medicines for treatment in areas with therapeutic gaps [18, 19].

However, like any other cost containment measure, there are drawbacks. Table 1 summarize the advantages and disadvantages of RSAs [20–22].

**Table 1:** Advantages and disadvantages of risk-sharing agreements.

	Advantages	Disadvantages
Patients/society	+ Access to innovative drugs	− Risks that the drug will not bring the expected benefits
	+ More treatment options and potential health improvements	− Termination of access to the drug at the end of the agreement
		− Data protection
Service providers	+ Expanding knowledge and improving the treatment of the disease	− Costs of implementing and monitoring the agreement
	+ Access to innovative drugs	− Digitalization of data and patient monitoring is a costly burden
	+ Reduce uncertainty about efficiency	− Complexity of managing multiple agreements
	+ Limit costs afterwards	
Payers	+ Collect additional evidence	− Difficulties in determining efficiency
	+ Uncertainty management (efficiency/budget)	− Lack of an integrated information system that collects data at the federal and regional levels
	+ Therapy aimed at patients with potential benefits	
Pharmaceutical companies	+ Access of innovative drugs to the market	− Implementation and monitoring costs/bureaucracy
	+ Increased drug efficacy for the target patient	− Risk of not demonstrating the proposed effectiveness
	+ Rewards for innovation	− Financial uncertainty, depending on the type of agreement
	+ Confidential terms of the agreement	− Biased selection of patients with the worst prognosis

These agreements are able to provide patients with access to innovative medicines in the face of uncertainty about their clinical benefit and cost-effectiveness due to still limited and/or immature evidence, identify groups of patients for whom the drug is most effective, and reduce the risk of unnecessary costs to payers when reimbursing them [23–26].

For payers, for example, the state body, the holder of its regional budgets, the insurer, the head physician, etc. On the one hand, they may face political criticism for any loss of public welfare if the product is subsequently found to be cost-effective. On the other hand, they face financial consequences and criticism for any loss of public welfare if the product is subsequently found to be cost-effective. On the other hand, they face financial consequences and criticism for any losses if the product is not cost-effective and/or little to no benefit to patients. In these circumstances, it may also serve as a poor benchmark as a standard of care for comparison with future treatments.

### 3.2 Examples of organizations, committees, performance evaluation standards

Clinical trials are often driven by clinical events that may not have standard definitions and may be misinterpreted. Funders need a method to reduce the impact of this potential variability on the conclusions drawn from the results data. Thus, the interpretation of the results of the study and the effectiveness of therapy is the most important problem on the way of manufacturers, pharmaceutical companies, etc.

For example, to evaluate efficacy in the United States, the following have been created: A Data Monitoring Committee (DMC) and Clinical Endpoint Committee (CEC), respectively (Table 2).

**Table 2:** Examples of organizations, committees, standards for performance evaluation.

	Data monitoring committee (DMC)	Clinical endpoint committee (CEC)
Primary goal	Provide recommendations to the sponsor based on intermediate comparative data to adjust the protocol, stop the study or continue unchanged	Provide endpoint evaluation
Focus during the study	Review of the safety and, in some cases, the effectiveness of the study and the conduct of the study	Review specific medical events by examining baseline data and determining whether events meet protocol-established criteria
Sources	Data summarized in lists/tables, which are usually created after the collection of initial data by biostatisticians	Raw data, such as statement summaries, CT scans, etc., related to specified endpoints
Participation in the preliminary study	Preliminary study participants may be asked to contribute to the development of the protocol prior to the start of the study	Some trials may use trial committees to verify patient eligibility prior to enrollment

Sometimes referred to as the Data and Safety Monitoring Board (DSMB), an independent group of experts who track data on patient safety and treatment effectiveness during clinical trials.

The CEC is a centralized decision-making body on safety and efficacy endpoints. The purpose of the CEC is to standardize results and optimize data quality, ultimately contributing to further successful use. CEC solves the problem of identifying efficacy and safety endpoints that are scientifically measurable, objective and justified.

The above Clinical Point Committee is an independent group of clinical, diagnostic experts who:

– Centrally analyze and classify possible safety and/or efficacy endpoints in a blind and confidential manner;
– Determine whether potential endpoints meet protocol definitions and endpoint criteria;
– Provide standardized deliverables for statistical analysis;
– Classify events as related to a device or research procedure [27].

In addition to data relating to specific endpoints, the CEC also considers general data: serious side effects, reports of death and side effects that could potentially become endpoints of clinical trials. In the case of medical devices, CEC inspection also includes serious, unforeseen side effects of the device and its shortcomings.

### 3.3 Types of agreements

**Figure 3:**
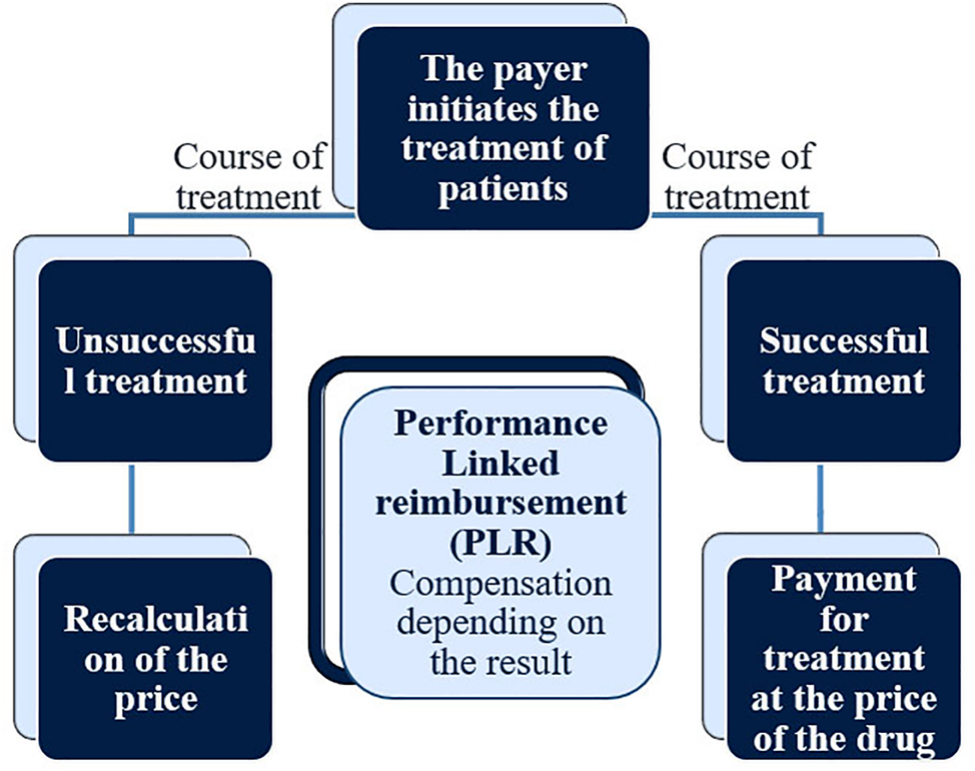
Performance linked reimbursement.

**Figure 4:**
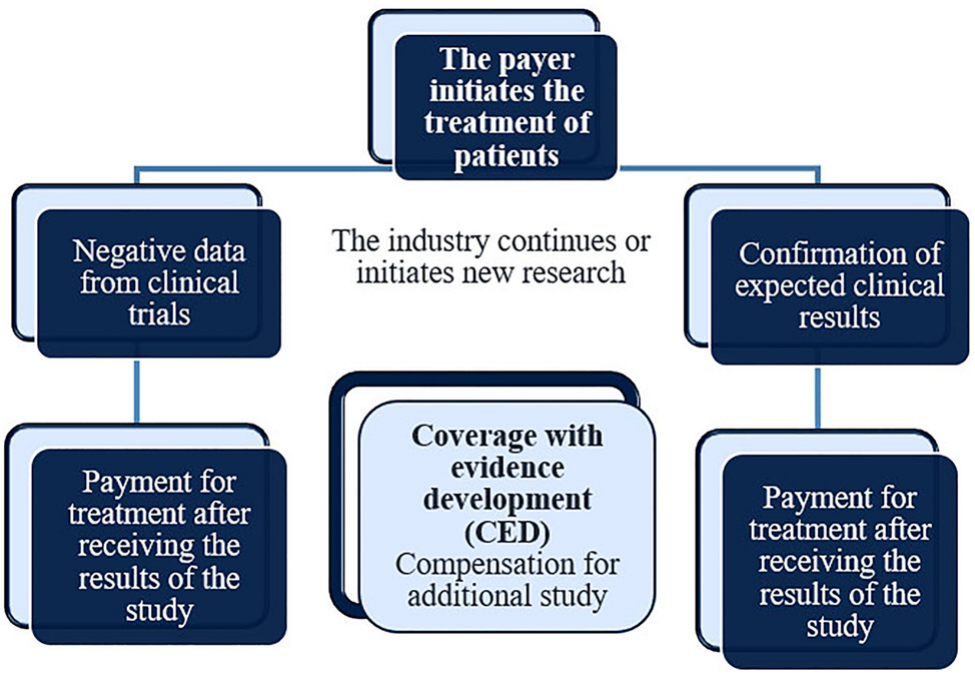
Coverage with evidence development.

**Figure 5:**
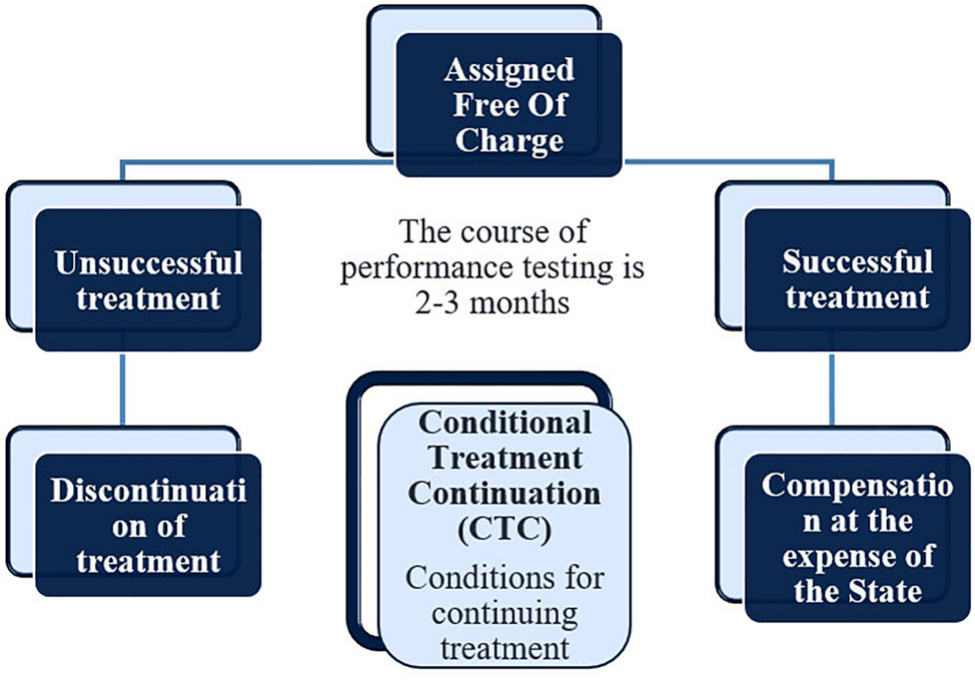
Conditional treatment continuation.

Examples of risk-sharing agreements include:

– PLR (Performance Linked reimbursement) – Reimbursement depending on the result (Figure 3);
– CED (Coverage with evidence development) – Reimbursement for additional study (Figure 4);
– CTC (Conditional Treatment Continuation) – Conditions for continuing treatment (Figure 5).

### 3.4 The importance of registries in implementing RSAs

One of the key steps required for the effective implementation of RSAs is the creation and/or adaptation of information systems that allow the collection of data on the use of medicines. This collection should preferably be carried out in an automated manner and using existing information systems along the patient’s route in order to minimize the additional administrative burden on health workers and maximize efficiency within institutions. Thanks to these features, the system will be able to issue reports in real time to provide immediate and continuous access to monitoring the use of drugs and the clinical situation of patients [1].

The first step is to define a matrix containing the minimum necessary parameters for collection, for example, in an observational study or a clinical trial. This choice of variables must necessarily reflect the type of disease and take into account the nature of the various agreements that may be concluded, as well as the flow of information that can potentially be established between the various actors involved (payer, supplier and pharmaceutical company) (Table 3).

**Table 3:** Minimum data required for the implementation of risk sharing agreements.

Patient information	− Unique patient identifier (anonymized)
	− Detection of the disease (diagnosis)
	− Mutational status (presence of concomitant mutations/diseases)
Information about drugs	− Main/related drug
	− Injected amount (dosage)
	− Dosage form
	− Introduction date
Treatment information	− Treatment status (not started/ongoing/finished)
	− Evaluation of response to treatment (complete response/partial/death/disease progression)
	− Evaluation date (intermediate/final)
	− Treatment end date
	− Reason for discontinuation of treatment

### 3.5 Creation of databases on the basis of cooperation of stakeholders contributes to the improvement of the health care system

The creation of databases contributes to an increase in the use and collection of medical data.

**Figure 6:**
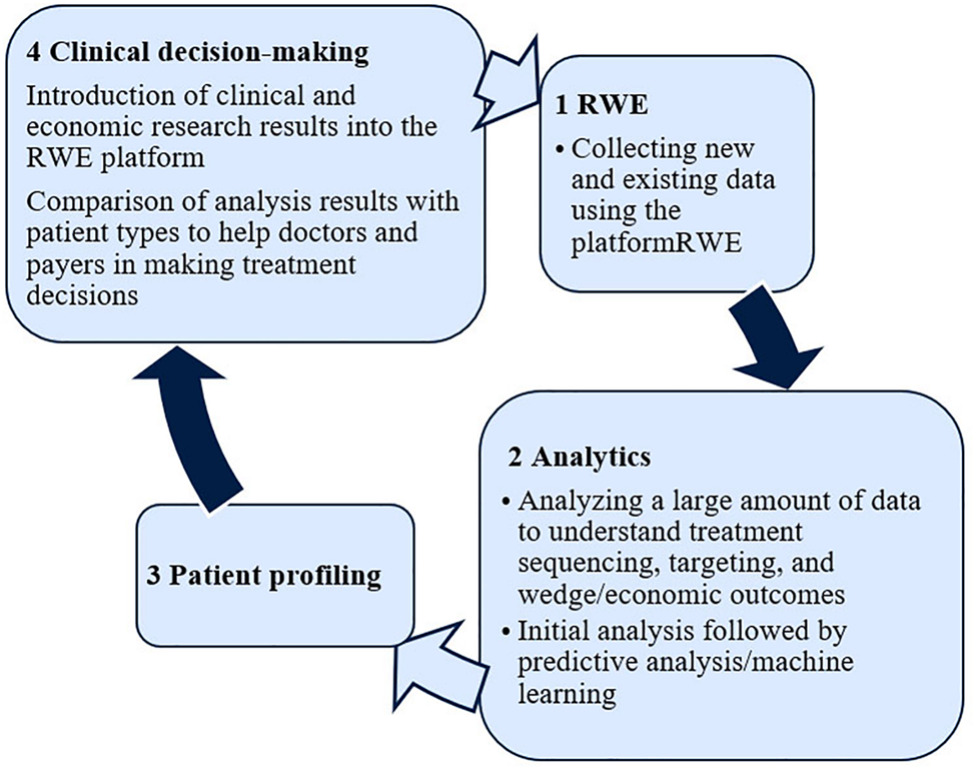
Database creation and use cycle.

The capabilities of AI make it possible to apply it at almost all stages of the cycle (Figure 6) starting from the formation of databases, analytics of incoming information, ending with the formation of a sample of patients, as well as structuring and aggregation of the collected data.

Learn more:

– AI can be trained to update regulatory documents, which in turn will allow for a comprehensive assessment of health technologies, registration in a shorter time and, therefore, eliminating the delay in access;
– Thanks to artificial intelligence, the analysis of Real-world data (RWD) and real-world evidence (RWE) data will be facilitated;
– Creation of the financing models, calculation of risks;
– Also, AI will speed up the process of forming a sample of patients, will provide a better selection that meets certain requirements.

### 3.6 Artificial intelligence

The digitalization of healthcare and medicine, the growing availability of electronic resources (medical records) encourage clinical researchers to use advanced methodologies in the field of artificial intelligence (AI) and big data analytics to use existing large medical databases. In hospitals, clinics, etc., the application of machine learning to access and analyze unstructured, free textual information gathered in a common database (e.g., drug safety, patient history, drug adverse reactions, interactions, errors, therapeutic outcomes, and pharmacokinetic effects) can be an important tool for improving patient care and conducting assessments of the efficacy, safety, and comparative efficacy of available drugs in real time [28].

Electronic medical records should contain information about prescriptions, treatment results, socio-demographic characteristics, previous concomitant diseases, test results, differential diagnosis, procedures, genetic background, signs and symptoms, family history and lifestyle habits [28].

The costs and time associated with manual data collection far outweigh the costs associated with using automated tools. The combination of machine learning and AI has proposed new descriptive and predictive ideas in clinical populations [29], patient management [30] and pharmacovigilance [31], and also shows great promise for creating computer support for clinical decisions [32].

### 3.7 Structuring information with AI

**Figure 7:**
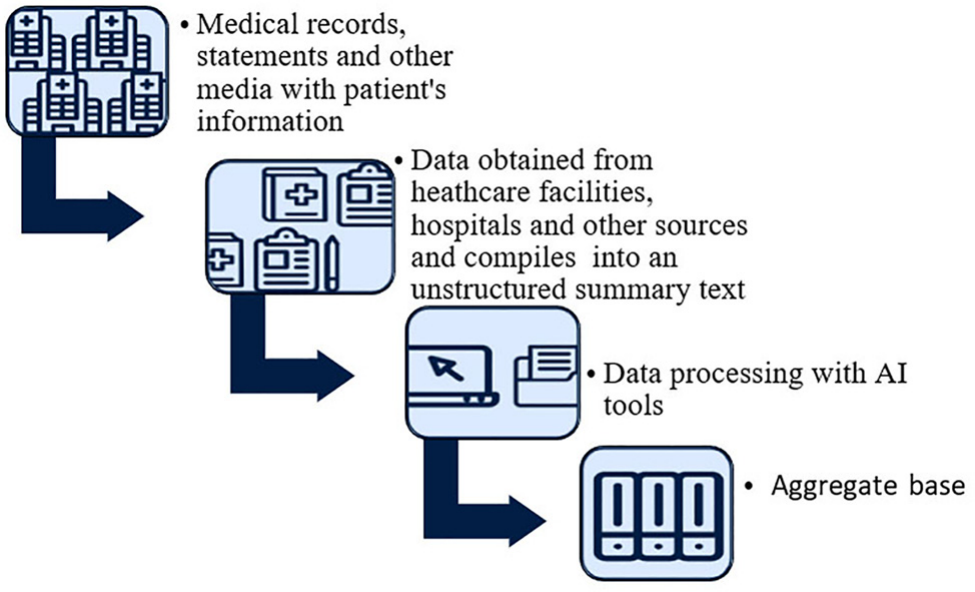
AI processing and aggregation.

Figure 7 presents the proposed scheme for the use of AI. Its capabilities allow not only to process and structure unstructured information, but also to collect, aggregate into certain databases.

The next emerging problem is interoperability – the availability and exchange of data. Existing concerns of politicians, hospital executives, and local regulators are often based on misconceptions about data privacy and security, limiting data availability and sharing between professionals and clinical researchers. The application of big data analytics in healthcare is an inevitable reality that needs solid rules to facilitate the availability and sharing of data [28].

### 3.8 Risks and limitations

The scope of the opportunities, industries affected and possible applications seem unlimited. The development of this technology, combined with the ever-increasing amount of data available that plays a key role, seems to be leading to a new era of artificial intelligence. But, as with any new technology, there are new risks and challenges associated with AI.

Processing large amounts of data, even using cloud services, can be costly. Developing a specialized AI-based system is expensive. Next question is privacy. Members of the medical community are concerned about data privacy due to the use of artificial intelligence and machine learning. Organizations that upload data to cloud services need special information protection tools, for example, encryption tools [14]. Issues of ethics, equality, bias, reliability of decisions made or proposed by AI are just some of the topics that need attention. Transparency of algorithms and growing cyber risks are also challenges that need to be addressed.

### 3.9 Additional features and benefits of artificial intelligence

From its ability to process large amounts of data to automating certain repetitive and burdensome risk management steps, AI can allow risk managers to respond more quickly to new and emerging risks. By operating in real time and with some predictive capabilities, risk management can reach a new level in supporting more effective decision-making [15].

AI is an umbrella term covering multiple methods to work with data.

An artificial neural network is mainly a mathematical apparatus, although sometimes elements of logic are found in various paradigms of neural networks.

A neural network is a mathematical model, the prototype of which is the central nervous system of a person or animal.

Fuzzy logic, fuzzy set theory, fuzzy reasoning, soft calculations are all close or closely related concepts related to a higher level of central nervous system work than artificial neural networks. Methods of fuzzy logic are used in experimental systems, object control systems.

Fuzzy logic is more related to the qualitative assessment of the analyzed processes and phenomena and decision-making based on this qualitative assessment.

This AI method could be used in pattern recognition, forecasting, classification, clustering and optimization tasks.

Within the framework of this group of methods, the concept of not individual, but collective intelligence is considered.

Evolutionary modeling is useful when the solution search space is so large and complex that traditional and simpler methods are simply unable to perform a global search for a solution or are capable, but it will take an unacceptably long time.

An expert system is an artificial analogue of a decision-maker, or at least an expert consultant of a subject area. The structure and logical and mathematical apparatus of the expert system are determined, first of all, by its purpose and subject area. The solutions proposed by the system can be developed using various output mechanisms. The closest analogue to the human inference mechanism is the apparatus of fuzzy logic and fuzzy set theory.

Machine Learning is a whole class of artificial intelligence methods. All of them imply the solution of problems not directly, but through preliminary training both before and in the decision-making process.

All these features could be implemented in medical data processing to predict results of the treatment, to analyse medical data and, for example, classify if the estimate treatment goals were reached.

The key benefits for risk management will be related to the following areas.

– Dataprocessing: the use of not only structured, but also unstructured data in huge volumes; text mining, database search, social network analysis and anomaly detection, which are combined with large-scale predictive models [14].
– Increased efficiency: reduce costs by automating day-to-day assistance and guidance in risk management processes.
– Real-time forecasting capabilities: awareness of new risks, increased number of risk prevention recommendations, faster response in critical situations.
– Business decisions: more effective decision-making due to greater (predictive) insight and visibility of risks (including for senior management).

Existing constraints are related to risk management, as well as to data sets, research, modelling and monitoring. Decisions that were previously made mainly “on instinct” or by benchmarking will become data-driven and systematic [15].

## 4 Discussion

Unfortunately, the hype surrounding risk-sharing agreements always seemed to outpace achievements. Despite the abundance of articles, presentations and discussions on this topic, real experience has shown that their implementation is not easy. Barriers include high implementation costs, measurement issues, lack of trust between payers and product manufacturers, and lack of a suitable data infrastructure. For many potential partners, negotiating price reductions has proven to be easier and less risky than entering into risk-sharing agreements.

However, in this work, we reflected the advantages and possible limitations, schemes for using this technology, coupling with artificial intelligence, were proposed. The main advantages that can be key in reducing the ever-increasing prices and costs of health care were shown.

The topic of this work is subject to more in-depth analysis and further developments on potential implementation in the health care system.

## 5 Conclusions

Considering the topic of using artificial intelligence and machine learning in conjunction with risk-sharing agreements, we can conclude that the introduction of these technologies will help not only pharmaceutical companies to provide innovative drugs in a shorter time, but also contribute to reducing, at least slowing down, the growth of health care costs.

When writing the work, perspective technologies were considered and analyzed: RSAs and AI. PLR, CED, CTC types of risk-sharing agreements, advantages and limitations of promising technologies were also analysed. As a result, it is shown that risk-sharing agreements could be beneficial for all stakeholders, but multiple challenges are still present to overcome.

This paper described the role of IT, in particular the capabilities that artificial intelligence can provide. Namely, AI can search for patients to participate in clinical trials. Using machine learning skills, you can teach artificial intelligence to analyze genetic information to identify suitable patient populations for trials and determine, for example, the necessary sample size. Probably, the main tasks of the application of artificial intelligence and machine learning are to speed up processes and make it easier for people to find and analyze a huge amount of data. Speeding up will result in reducing the time to search for a sample, to conduct tests, to analyze the data obtained – all this will allow pharmaceutical companies to quickly and earlier bring the drug to the market. That will ultimately be reflected in the economic component of the sphere. After all, early launch and registration allow to start treating patients earlier, thereby reducing various costs, both state (for example, loss of GDP due to disabled citizens, maintenance of patients, etc.) and costs for pharmaceutical companies.

Returning to machine learning, it is impossible not to mention one of the main qualities – imitation of the work of the human brain, but at the same time it is able to make a decision much faster and more accurately. The creation of an algorithm will be able to contribute to the acceleration of data analysis, it will allow to predict the results of research with greater accuracy.

This research work brings scientific novelty in terms of the combination of promising technologies that today remain promising, unfortunately, only on paper. The results of the work can be applied in future economic research, including other industries not related to pharmacy.
